# The copper resistance mechanism in a newly isolated *Pseudoxanthomonas spadix* ZSY‐33

**DOI:** 10.1111/1758-2229.13163

**Published:** 2023-06-16

**Authors:** Hongjie Wang, Siyao Zhang, Jing Zhang

**Affiliations:** ^1^ Hebei Key Laboratory of Close‐to‐Nature Restoration Technology of Wetlands Hebei University Baoding People's Republic of China; ^2^ Institute of Xiong'an New Area Hebei University Baoding People's Republic of China; ^3^ School of Eco‐Environment Hebei University Baoding People's Republic of China; ^4^ College of Life Science Hebei University Baoding People's Republic of China

## Abstract

Resolving the heavy metal resistance mechanisms of microbes is crucial for understanding the bioremediation of the ecological environment. In this study, a multiple heavy metal resistance bacterium, *Pseudoxanthomonas spadix* ZSY‐33 was isolated and characterized. The copper resistance mechanism was revealed by analysis of the physiological traits, copper distribution, and genomic and transcriptomic data of strain ZSY‐33 cultured with different concentrations of copper. The growth inhibition assay in basic medium showed that the growth of strain ZSY‐33 was inhibited in the presence of 0.5 mM copper. The production of extracellular polymeric substances increased at a lower concentration of copper and decreased at a higher concentration of copper. Integrative analysis of genomic and transcriptomic, the copper resistance mechanism in strain ZSY‐33 was elucidated. At a lower concentration of copper, the Cus and Cop systems were responsible for the homeostasis of intracellular copper. As the concentration of copper increased, multiple metabolism pathways, including the metabolism of sulfur, amino acids, and pro‐energy were cooperated with the Cus and Cop systems to deal with copper stress. These results indicated a flexible copper resistance mechanism in strain ZSY‐33, which may acquire from the long‐term interaction with the living environment.

## INTRODUCTION

Copper is an essential micronutrient for bacteria. The majority of living organisms require copper as a redox cofactor for a variety of vital biological processes (Rubino & Franz, 2012), such as cytochrome oxidase, laccase, superoxide dismutase, plastocyanin, and nitrite reductase (Arguello et al., 2013; Sousa et al., 2019). Excess copper, however, is also cytotoxic (O'Hern & Djoko, 2022; Trevors & Cotter, 1990). Copper ions generate reactive oxygen species (ROS) through auto‐oxidation or Fenton‐like reactions (Koppenol, 2001), which lead to lipid peroxidation, protein oxidation, and DNA damage (Pytharopoulou et al., 2008; Rehman et al., 2019; Zhang et al., 2017). Moreover, excessive copper ions can cause damage to proteins (Higgins et al., 2020). In the process of long‐term evolution, bacteria have evolved a variety of strategies to counteract copper toxicity (Ladomersky & Petris, 2015; Pal et al., 2022; Voica et al., 2016). These include copper efflux systems, extracellular sequestration, intracellular accumulation, and enzymatic detoxification (Andrei et al., 2020; Giachino & Waldron, 2020; Gracioso et al., 2021; Mathivanan et al., 2021b; Yin et al., 2019).

To maintain the homeostasis of copper ions, genes involved in copper efflux systems are tightly regulated (Giachino & Waldron, 2020). In Gram‐negative bacteria, a large number of transmembrane copper efflux systems have been identified. For example, the Pco, Cus, and Cut systems in *E. coli* (Grass & Rensing, 2001) and the Cop system in *Pseudomonas aeruginosa* (Novoa‐Aponte et al., 2020; Quintana et al., 2017). The Cus system is a heavy metal resistance‐nodulation‐cell division (RND) efflux system composed of *cusCFBA* operon (Delmar et al., 2015) and regulated by *cusRS* genes (Munson et al., 2000). The function of the Cus system is to collect Cu^+^ from the periplasmic and cytoplasm and excrete them using chemical reactions with methionine residues, which is proton‐motived (Delmar et al., 2013; Mealman et al., 2012; Pal et al., 2022). The Cop system (P‐type ATPase) located in the inner membrane is ATP promoted and responsible for the transportation of metal ions from the cytoplasm to the periplasm (de Freitas et al., 2019). These systems play a key role in copper efflux and protect cells from copper cytotoxicity.

Extracellular sequestration is the most widely used method for heavy metal removal, which can efficiently decrease the toxicity of heavy metals to microorganisms. Heavy metal ions are accumulated by the combination with extracellular polymeric substances (EPS), metallophores (Mohr et al., 2021; Sun et al., 2023; Wichard et al., 2008), or the formation of heavy metal complexes with cellular metabolites. In addition, copper ions can be converted to zero‐copper by a copper‐resistant bacterium (Gracioso et al., 2021). The metal ions promote the production of EPS, which provides binding sites such as alcohols, carboxylic acids, and aliphatic amines for metal ions (Rathi & Yogalakshmi, 2021). The formation of intracellular or extracellular metal nanoparticles, especially metal sulfide, is a common phenomenon, which is a convenient method for microorganisms to decrease the concentration of heavy metals in the environment to reduce their toxicity (Park & Faivre, 2022).

The genus *Pseudoxanthomonas* belongs to the family *Xanthomonadaceae*, which can be found worldwide in freshwater lakes and streams, soil, wetlands, and polluted sites (Oren, 2014). Genus members of *Pseudoxanthomonas* are a valuable source for further studies focused on the degradation of refractory organics (Wang et al., 2011), the reduction of nitrite and nitrate (Sun et al., 2021), and the tolerance of heavy metals (Mohapatra et al., 2018). *Pseudoxanthomonas* was the most abundant member in the sludge polluted by high concentrations of Cu^2+^ (Jiang et al., 2020). However, the mechanisms of *Pseudoxanthomonas* in copper resistance have not been elucidated.

In the current study, a novel copper‐tolerant bacterium was isolated from the Baiyangdian wetland, and the phenotypic traits were described. The minimum inhibition concentration (MIC) of copper as well as other heavy metals was determined, and the removal rate of copper was evaluated. The copper resistance mechanism was analysed by combining inductively coupled plasma‐mass spectrometry (ICP‐MS), scanning electron microscopy (SEM), and genomic and transcriptomic methods. Based on these results, we hypothesized that the copper‐resistant mechanism of strain ZSY‐33 was quite different under the stress of different concentrations of copper. Overall, this study drew a comprehensive picture of how strain ZSY‐33 defends itself against the negative impact of copper and widened the understanding of copper resistance mechanisms of the genus *Pseudoxanthomonas*.

## EXPERIMENTAL PROCEDURES

### Isolation and cultivation of strain ZSY‐33

Strain ZSY‐33 used in this study was isolated from sediment samples collected from the Baiyangdian Lake (116°17′04.956″ E, 38°06′58.384″ N), Baoding City, Hebei Province, China. About 0.5 g sediment samples were incubated with 10 mL sterile PBS (10 mM, pH = 7.2) overnight. To isolate a single colony, spread plating was performed after gradient dilutions. Colonies were isolated with R2A medium, which contained 0.5 g yeast, 0.5 g peptone, 0.5 g glucose, 0.5 g casein hydrolysate, 0.5 g soluble starch, 0.3 g sodium pyruvate, 0.3 g KH_2_PO_4_, and 0.024 g MgSO_4_. The corresponding solid medium contained 15 g/L agar.

The purified isolate was cultured at 28°C in the basic medium, which contained 5 g peptone, 1 g yeast extract, and 5 g NaCl in 1000 mL deionized water. The screening of copper‐resistant strains was performed on solid basic medium amended with 0.5 mM Cu^2+^. The growth curve of strain ZSY‐33 was constructed in basic medium at 28°C with shaking at a speed of 150 rpm. To determine the effect of divalent copper on the growth of strain ZSY‐33, a final concentration of 0.05, 0.25, 0.5, 0.6, 0.7, and 1.0 mM copper sulfate was amended into the medium, respectively. A culture without heavy metals was used as the control. The absorbance of bacterial culture at 600 nm (OD_600_) was measured at 3 h intervals from 0 to 60 h.

### Phylogenetic analysis

The water‐boiling method was used to extract the genomic DNA of the isolates. Universal primers 27F (5'‐AGAGTTTGATCCTGGCTCAG‐3′) and 1492R (5’‐GGCTACCTTGTTACGACTT‐3′) were used to amplify the 16S rRNA gene sequence. To determine the phylogenetic position of ZSY‐33, the BLAST programs (https://www.ncbi.nlm.nih.gov/) were used to analyse the 16S rRNA gene sequence, and the phylogenetic tree was constructed with MEGA X (Kumar et al., 2018).

### Determination of copper MIC and removal rate of stain ZSY‐33

To determine the copper MIC of stain ZSY‐33, mid‐log‐phase culture was inoculated in the basic medium (100 mL) containing 0, 0.05, 0.1, 0.15, 0.2, 0.25, 0.3, 0.4, 0.45, 0.5, 0.55, 0.6, 0.65, 0.7, 0.75, 0.8, 0.9, 1.0, and 1.1 mM copper sulfate (CuSO_4_) respectively. All samples were set in duplicate. The growth of bacteria was monitored by measuring the OD_600_ after incubation for 24 h, at 28°C and 150 rpm. The MIC value was defined as the minimum copper concentration to inhibit the growth of strain ZSY‐33 after 24‐h incubation. And the EC_50_ value of copper was calculated.

To determine the removal rate of copper from the medium and the distribution of copper in strain ZSY‐33, bacteria were grown in basic medium (100 mL) containing 0.025, 0.5, 0.1, 0.2, 0.25, 0.3, 0.4, and 0.5 mM copper sulfate (CuSO_4_), respectively at 28°C for 24 h. Cultures without inoculum were conducted as the control group. After incubation, cells were collected, centrifuged (10,000 *g* for 10 min), and filtered with 0.22 μm filters to remove cellular debris. The copper concentration in the supernatant was determined. To figure out the distribution of copper, EDTA was used to extract the copper adsorbed on the cell (Huang et al., 2019). After being washed three times using PBS (10 mM pH = 7.2), the copper ions were extracted through sterilized EDTA (25 mM) by agitating at 150 rpm for 30 min. Intracellular copper was released by digestion using 70% nitric acid for 30 min. The EDTA‐extracted copper ions were defined as extracellular biosorption, and the non‐EDTA‐extracted copper ions were considered intracellular bioaccumulation. The concentration of Cu was detected by ICP‐MS (NexION 350X, PerkinElmer, USA), with the following sets, radio frequency power 1300 W, atomized gas (argon gas) flow rate 1 L/min, auxiliary gas (argon gas) flow rate 1.2 L/min. The removed copper (m) and copper removal percentage (p) from the cultured medium was determined by the following equation:

m = C_0_ − C_1_,

p = m/C_0_ × 100%,

where C_1_: copper concentration in the medium incubated after 24 h. C_0_: initial copper concentration.

### Analysis of hydrogen sulfide generation

To detect the production of H_2_S by strain ZSY‐33, cells were inoculated in 100 mL basic medium with different concentrations of copper sulfate and incubated in a flat‐bottomed flask for 24 h (28°C, 150 rpm). The wet paper strips with lead acetate were fixed on the top of the flask. The generation of lead sulfide (black precipitation) was taken as the detection mark of H_2_S production, and this analysis was conducted by visual comparison (Ma et al., 2021).

### Preparation for scanning electron microscopy

Strain ZSY‐33 was inoculated in basic medium amended with 0, 0.25, and 0.5 mM Cu^2+^, respectively (28°C, 150 rpm). After incubation, culture (OD_600_ = 0.5–0.8) was centrifuged (2400 *g* for 5 min) and washed twice with PBS. The cells were fixed with glutaraldehyde (2.5%) for 2 h, and the cells were dehydrated step by step using gradient ethanol (30%, 50%, 70%, 80%, 90%, and 95%), each concentration for 15 min, and 100% ethanol twice for 20 min each. The cells were placed on the conductive adhesive tape, observed, and photographed by SEM.

### Genome sequencing and analysis

Wizard® Genomic DNA Purification Kit (Promega) was used to extract the genomic DNA of strain ZSY‐33. After purification, the extracted DNA was sequenced using a combination of PacBio RS II single molecule real time and Illumina sequencing platforms. The complete genome sequence was assembled using both the PacBio reads and Illumina reads. Low‐quality data were removed from the clean data, and the reads were assembled into a contig using a hierarchical genome assembly process and canu (Koren et al., 2017). Glimmer (Delcher et al., 2007), tRNA‐scan‐SE (Borodovsky & McIninch, 1993), and Barrnap were used for CDS prediction, tRNA prediction, and rRNA prediction, respectively. The predicted CDSs were annotated from NR, KEGG, COG, GO, Pfam, and Swiss‐Prot databases using sequence alignment tools such as HMMER, Diamond, and BLAST. All the analyses were performed based on the online platform provided by Majorbio Cloud Platform (www.majorbio.com) from Shanghai Majorbio Bio‐pharm Technology Co. Ltd.

### Transcriptional profiling of strain ZSY‐33 cultured with different concentrations of Cu^2+^

To further explore the copper resistance mechanism of stain ZSY‐33, transcriptomic analysis was performed. Strain ZSY‐33 was cultured in 100 mL basic medium amended with copper sulfate at a final concentration of 0, 0.25, and 0.5 mM, respectively, in triplicate. The culture medium of each group was collected at the exponential phase. Cells were collected after 18 h of incubation by centrifugation (12,000 *g* for 10 min at 4°C). The pellets were washed with PBS (10 mM, pH = 7.2), and total RNAs were extracted with Trizol reagent (Invitrogen Life Technologies). The Bioanalyzer 2100 system (Agilent) and NanoDrop spectrophotometer (Thermo Scientific) were used to determine the integrity and quality of extracted RNA (Liu et al., 2016). The sequencing library was then sequenced on NovaSeq 6000 platform (Illumina) (Wu et al., 2022) by Shanghai Personal Biotechnology Cp. Ltd.

After filtering out reads that contained adapters, low‐quality bases, or unknown bases, clean reads were aligned to the reference genome sequence of strain ZSY‐33. HTSeq (v0.9.1) was used to count the gene read as the original expression level of the gene. FPKM (fragments per kilobase of exon per million fragments mapped) was used to normalize the expression of genes in different samples. DESeq was conducted to analyse the differential expressed mRNA, transcripts with log_2_|fold change| > 1 and *p*‐value<0.05 were considered as differentially expressed mRNA (Yan et al., 2021). To investigate the functional genes responsible for different copper stress, the relative expression of differentially expressed genes (DEGs) was further analysed via heat‐map based on the log_2_|fold change|. In addition, KEGG annotations were used to analyse the enrichment of DEGs.

### Gene expression RT‐qPCR validation

RT‐qPCR analysis was performed to validate the RNA‐Seq data. Cells were harvested after 18 h of culturing in basic medium amending with 0, 0.25, or 0.5 mM Cu^2+^. Total RNA was extracted using Trizol regent (Invitrogen, United States). Synthesis of cDNA was done using the Transcriptor First‐Strand cDNA Synthesis kit (Roche, China) according to the manufacturer's instructions. SYBR Green I Master mix (Roche) was used to performing the real‐time PCR. The detailed genes and primer information were shown in Table S1. The 2^−ΔΔCt^ method was used to analyse the relative expression of per DEGs (Schmittgen & Livak, 2008). Each reaction was conducted in triplicate biological and technical replicates.

### Statistical analysis

Data are presented as means ± SD. One‐way analysis of variance with interaction was carried out using SPSS software to identify the differences in removal efficiency and the distribution of copper under varied copper concentrations. *p* < 0.05 indicates that the difference is statistically significant.

## RESULTS

### Screening and identification of heavy metal tolerant bacteria

**FIGURE 1:**
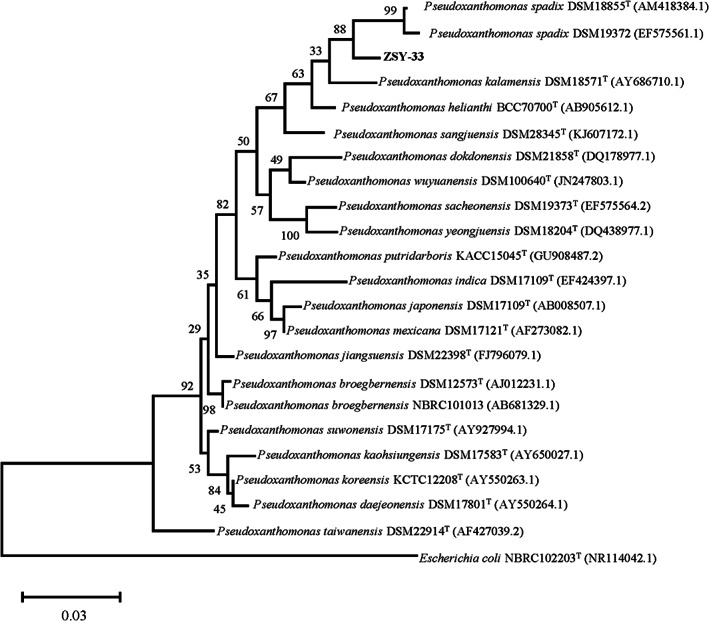
The consensus phylogenetic tree was constructed using the 16S rRNA sequences of strain ZSY‐33 and other related relatives in the genus *Pseudoxanthomonas* obtained from the GenBank (accession numbers of different 16S rRNA sequences were indicated after the species name). The tree was constructed by the maximum‐likelihood method and the bootstrap support value of 1000.

During screening heavy metal tolerance bacteria, strain ZSY‐33 was selected because it could grow in the presence of various heavy metals including copper, cadmium, arsenic, zinc, and nickel (Figure S1). The cells of strain ZSY‐33 were purified and cultured in the basic medium. The 16S rRNA gene sequence of strain ZSY‐33 showed a similarity of 97.89% with *Pseudoxanthomonas spadix* strain IMMIB AFH‐5. In addition, strain ZSY‐33 also clustered with *P. spadix* according to the phylogenetic analysis (Figure 1). Thus, strain ZSY‐33 was identified as a member of *P. spadix* and designated as ZSY‐33 in this study.

### Effect of Cu^2+^ on the physiological characteristics of strain ZSY‐33

**FIGURE 2:**
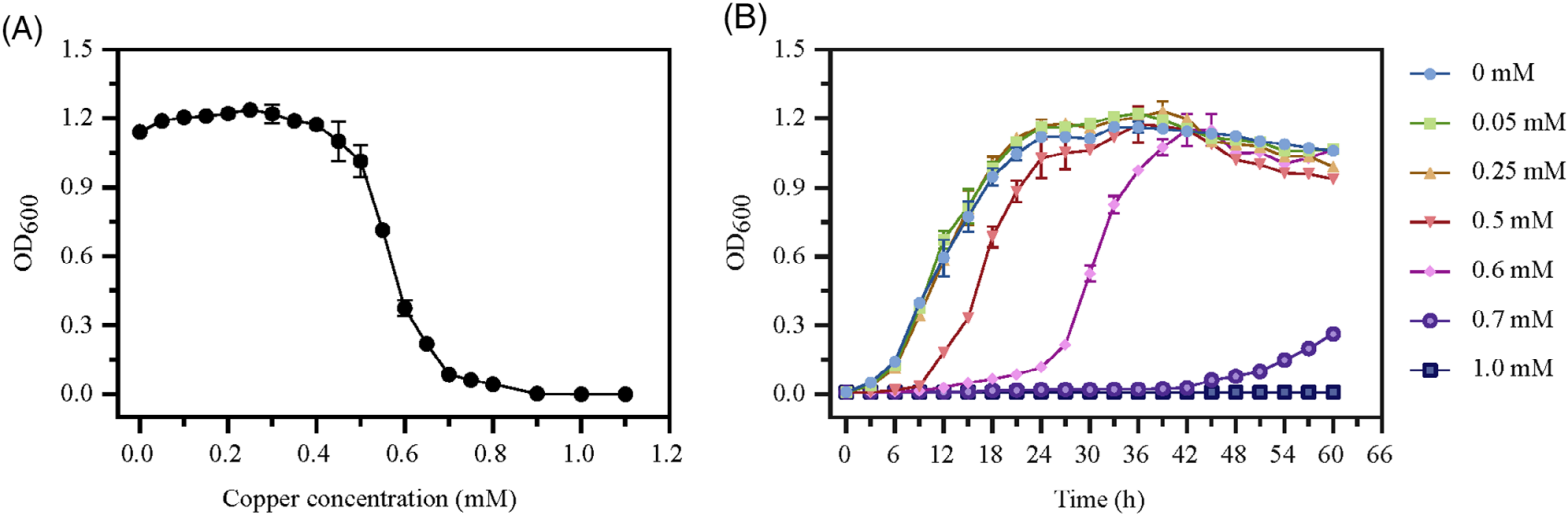
Growth characteristics of strain ZSY‐33 under copper stress. (A) The copper minimum inhibition concentration of strain ZSY‐33 was determined in the basic medium at 28°C and 150 rpm for 24 h. (B) Growth curve of strain ZSY‐33 in basic medium amending with different concentrations of Cu^2+^. The culture was conducted at 28°C and 150 rpm. The error bars indicate the SD from two different biological replicates.

*P. spadix* ZSY‐33 showed a lower Cu^2+^ MIC (0.9 mM; Figure 2A) in the basic medium, and a higher MIC (1.8 mM; Figure S2) in the Lysogeny Broth (LB) medium. The EC_50_ of copper in the basic medium and LB medium were 0.5625 and 1.374 mM (Figure S3), respectively. The influence of different concentrations of Cu^2+^ on the growth of strain ZSY‐33 in the basic medium was shown in Figure 2B. Supplementation with a concentration of Cu^2+^ lower than 0.25 mM had no noteworthy influence on the growth of strain ZSY‐33 compared with the control group. The growth of strain ZSY‐33 was significantly inhibited when the concentration of Cu^2+^ exceeded 0.5 mM. The toxic effect of high concentrations of copper on the bacteria was reflected in the retardation of the growth of strain ZSY‐33 and the delay of the appearance of the logarithmic phase.

**FIGURE 3:**
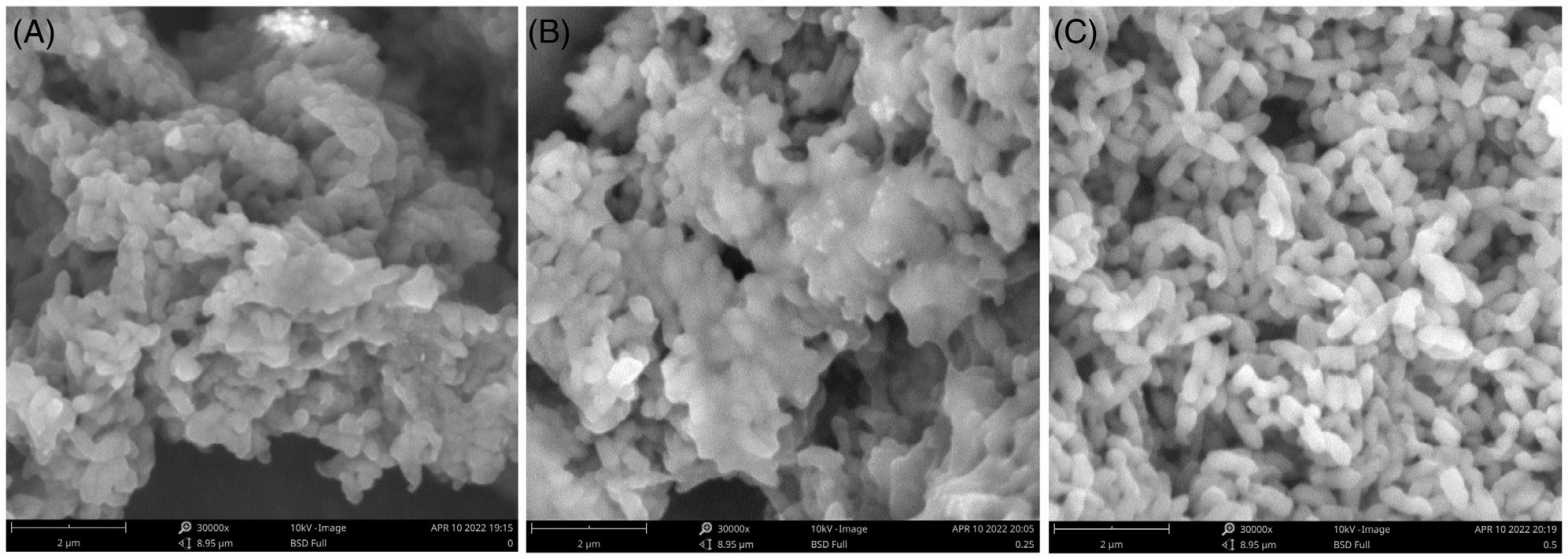
Scanning electron micrographs of strain ZSY‐33 exposed to different concentrations of copper (Cu^2+^). (A) The morphology of strain ZSY‐33 cultured without Cu^2+^; (B) the EPS surrounding strain ZSY‐33 increased under 0.25 mM Cu^2+^ treatment; (C) the EPS surrounding strain ZSY‐33 decreased under 0.5 mM Cu^2+^ treatment.

To further explore the effect of Cu^2+^ on the physiological characteristics of strain ZSY‐33, an SEM was employed. The results showed that the EPS around the cells were affected by the concentration of copper. In the 0.25 mM Cu^2+^ treatment, the EPS surrounding the cells was much thicker than that in the control group (Figure 3B), while it was decreased in the 0.5 mM Cu^2+^ treatment (Figure 3C). Lead acetate paper was used to detect the production of H_2_S. In the absence of Cu^2+^, strain ZSY‐33 could produce a large number of H_2_S (Figure S4). The production of H_2_S decreased in the samples treated with 0.025 mM Cu^2+^. In the samples supplemented with 0.1 mM Cu^2+^, no H_2_S was detected.

### Removal and distribution of copper ions

**FIGURE 4:**
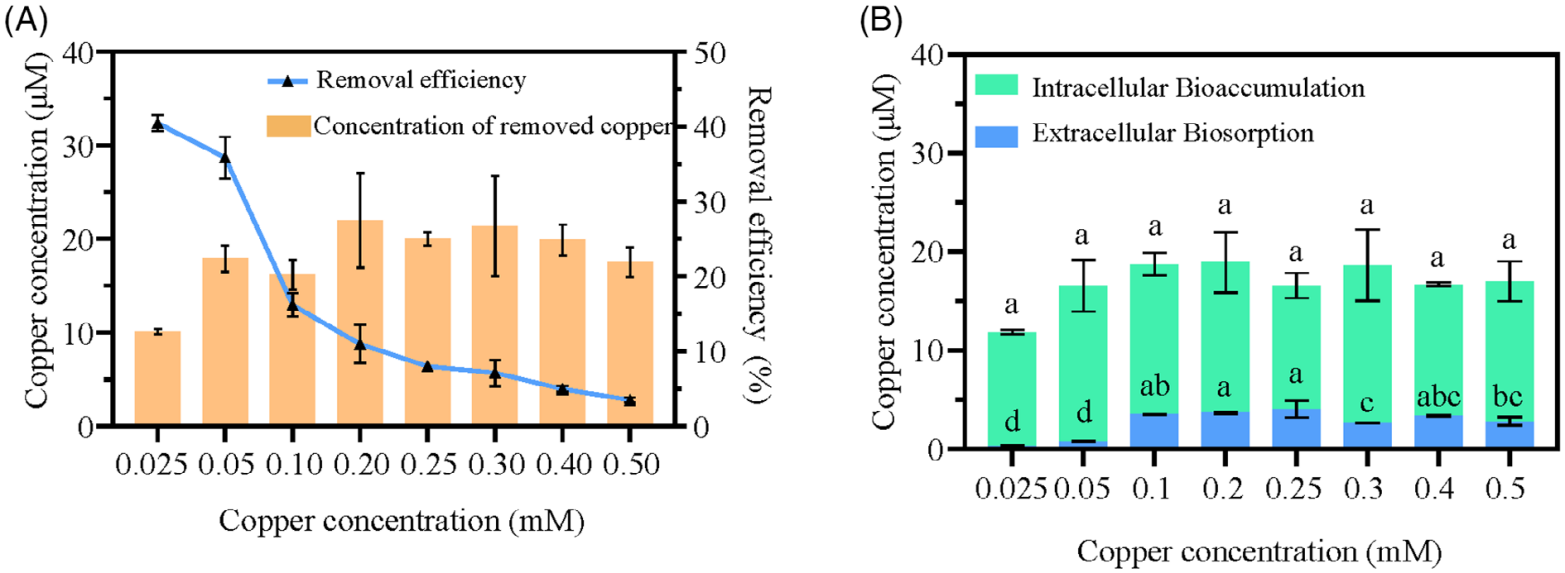
The removal rate of copper in the medium and distribution of copper in strain ZSY‐33 cultured in basic medium after 24 hours. (A) Cu^2+^ removal efficiency and the concentration of removed copper by strain ZSY‐33. (B) Distribution of copper (Cu) of strain ZSY‐33 cultured with different concentrations of Cu^2+^ in basic medium after 24 h. Intracellular accumulation and extracellular adsorption indicated the concentrations of copper inside the cells and absorbed on the cell surface, respectively. The error bars indicate the SD from two different biological replicates. Different superscripts indicated significant differences between treatments (*p*‐value < 0.05).

To explore the copper removal effect of strain ZSY‐33, a series of concentrations of Cu^2+^ were tested. The copper removal rate decreased with the increase of an initial concentration of Cu^2+^ (Figure 4A). The maximum removal rate of copper was 40.50% at an initial Cu^2+^ concentration of 0.025 mM, followed was 35.88% at an initial Cu^2+^ concentration of 0.05 mM. The removal rate of Cu^2+^ dropped to 8.02% and 4.98% when the initial concentration of Cu^2+^ was 0.25 and 0.5 mM, respectively. To further investigate the distribution of copper in the cell, the extracellular biosorption and intracellular bioaccumulation of copper were examined separately. Results showed that the extracellular adsorption reached a maximum of 4.04 μM at 0.25 mM Cu^2+^, while the intracellular bioaccumulation reached a maximum of 18.95 μM at 0.2 mM Cu^2+^ (Figure 4B). These results showed that strain ZSY‐33 preferred intracellular copper bioaccumulation to extracellular biosorption.

### Genome characterization and heavy metal resistance genes

To obtain a genetic basis for the Cu^2+^ resistance and biological removal in *P. spadix* ZSY‐33, the genome of *P. spadix* ZSY‐33 was completely sequenced. The complete genome of *P. spadix* ZSY‐33 contained only one circular chromosome and no plasmid. The overall GC content of the chromosome was 69.22%. By annotation, 3873 genes were predicted, 3813 of which were protein‐coding genes. In addition, 54 tRNA genes and 6 rRNA genes were identified on the chromosome of *P. spadix* ZSY‐33 (Table S2).

**FIGURE 5:**
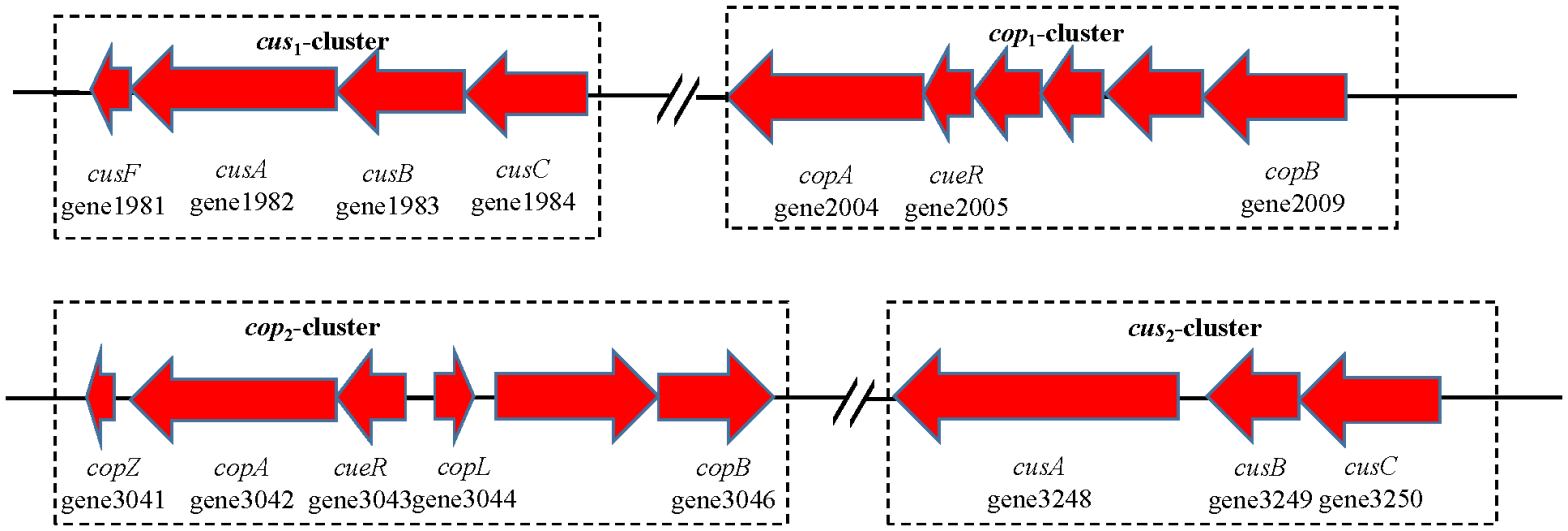
Orientation and product of putative gene clusters and operons involved in copper resistance detected in strain ZSY‐33.

Further analysis of the genome of *P. spadix* ZSY‐33, we found that it contained a large number of genes related to heavy metal resistance, including Cu, Cd, Zn, Co, Hg, As, and Ni. Typical metal resistance loci, such as *czcABCD*, *zntRBA*, *arsRBC*, *corAC*, and *merRTP*, which responded to multiple heavy metals were found (Figure S5). Besides, four different gene clusters related to the Cu‐resistant system, including two Cop systems and two Cus systems were found (Figure 5). The Cus1 system consisted of the gene *cusF* (*gene 1981*), *cusA* (*gene 1982*), *cusB* (*gene 1983*), and *cusC* (*gene 1984*), and the Cus2 system consisted of the gene *cusA* (*gene 3248*), *cusB* (*gene 3249*) and *cusC* (*gene 3250*). The Cop system consisted of *copA* (*gene 2004* and *gene 3042*), which encodes P‐type ATPases, *cueR* (*gene 2005* and *gene 3043*), *copB* (*gene 2009* and *gene 3046*), *copZ* (*gene 3041*), and *copL* (*gene 3044*).

### Differential gene expression under different concentrations of copper

To further understand genes and the molecular mechanisms involved in the copper stress of ZSY‐33, RNA‐seq was used for sequencing analysis. There were about 40 million clean reads obtained from nine samples. The Q30 values of all clear reads in the treatment of 0, 0.25, and 0.5 mM copper were greater than 94.70%, 94,94%, and 94.63%, respectively, illustrating the high reliability of the sequencing results. The results of the principal component analysis showed good parallel correlations and obvious differences for each set (Figure S6A). Compared with the control group, 210 genes (159 up‐ and 51 down‐regulated) and 1345 genes (668 up‐ and 677 down‐regulated) were significantly differentially expressed (FDR < 0.05, log_2_|fold change| ≥ 1) in the treatment of 0.25 mM and 0.5 mM Cu^2+^, respectively (Figure S6B).

### Transcriptomic profiling of copper resistance by strain ZSY‐33

**FIGURE 6:**
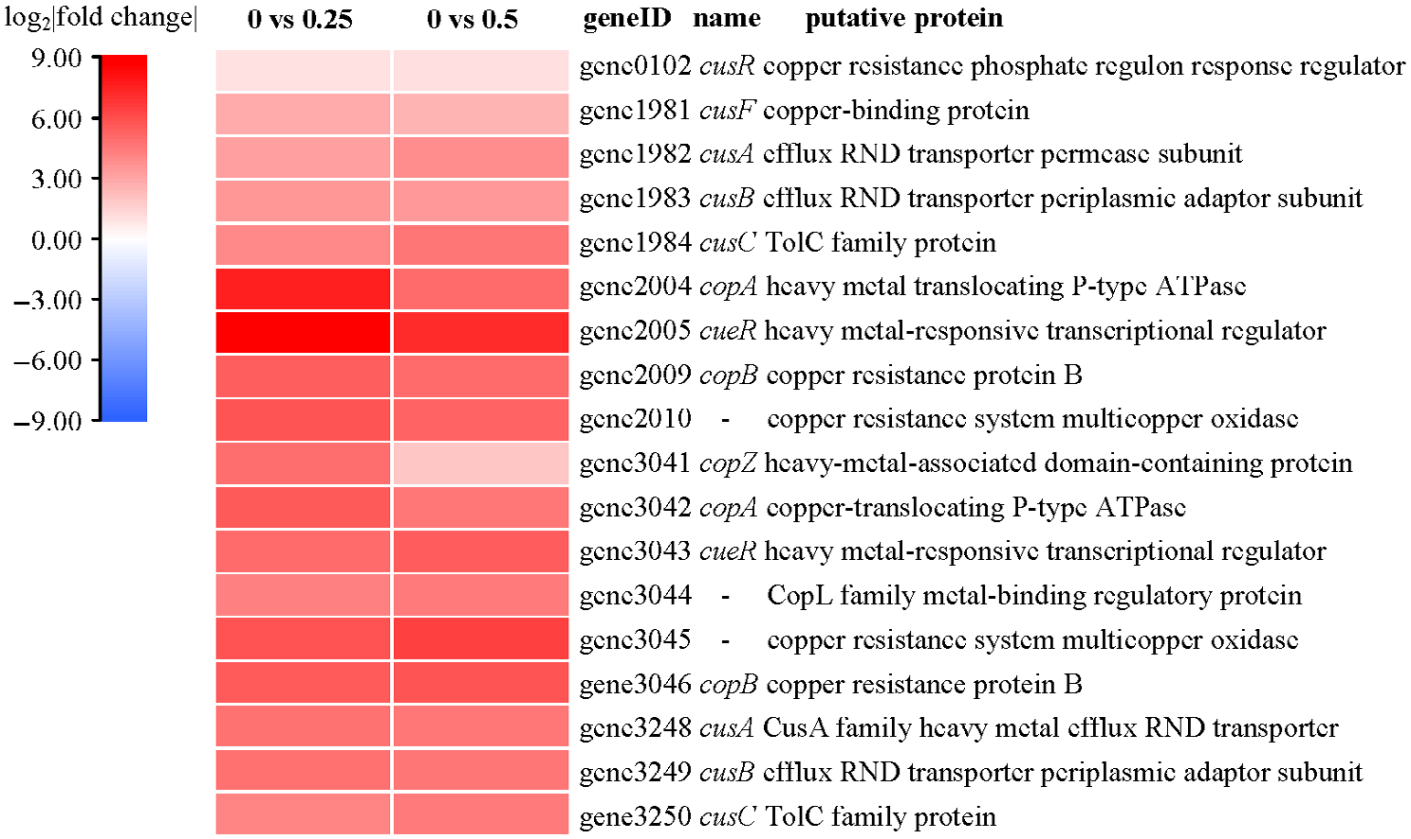
Differentially expressed genes (DEGs) related to copper resistance were shown using a heatmap. The column name “0 vs. 0.25” and “0 vs. 0.5” indicated DEGs in the treatment of 0.25 mM and 0.5 mM copper compared with a control group, respectively. Strain ZSY‐33 was harvested after 18 h incubation. Three biological repeats were set.

To investigate the functional genes responsible for copper stress, a heat map was used to further analyse the relative expression of DEGs based on the log_2_|fold change| (Figure 6). By comparing the DEGs in different concentrations of copper treatment, we found genes including *CusR*, *CueR*, *CusFABC*, and *CopZAB* were significantly up‐regulated. The expression levels of these genes were increased by 0.99 to 8.66 log_2_|fold change|. As shown in Figure 5, the copper resistance genes of strain ZSY‐33 consisted of genes encoding the Cus systems (CAB transporter) and the Cop systems (P‐type ATPase). Both copper resistance systems were significantly up‐regulated in response to copper stress. Among them, the expression of *cueR* (*gene 2005*), which encodes heavy metal‐responsive transcriptional regulator, was the most significantly up‐regulated gene under copper stress. The expression levels of *cueR* increased to 8.66 and 7.23 log_2_|fold change| when the Cu^2+^ concentrations were 0.25 and 0.5 mM, respectively, indicating that this gene may play a key role in the copper resistance of strain ZSY‐33.

### Analyses of DEGs enriched in the KEGG pathway

**FIGURE 7:**
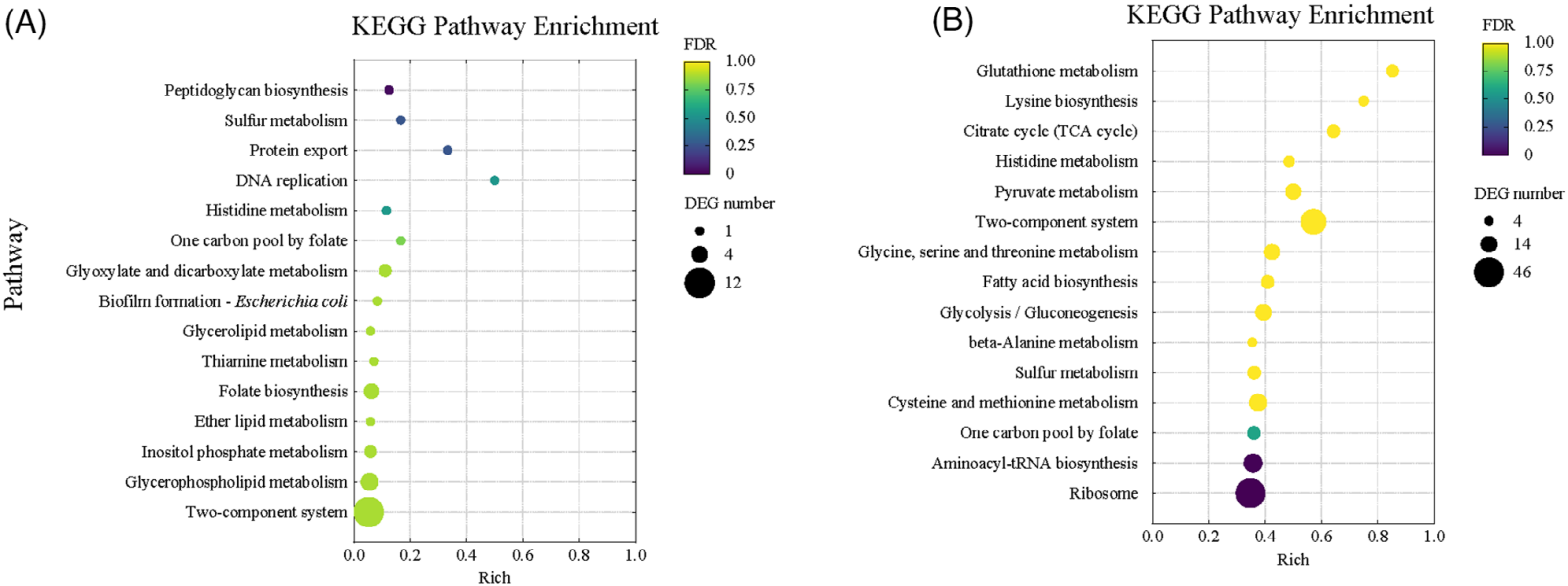
KEGG pathways enriched in strain ZSY‐33 under different concentrations of copper stress. The y‐axis showed individual KEGG pathways, while the x‐axis indicated the enrichment factor. The bubble size represented the number of DEGs involved, and the bubble colour indicated the enrichment degree of the pathway. Only the 15 most significant KEGG pathways were presented in this figure. Significant KEGG pathways enriched under 0.25 mM Cu^2+^ stress (A), and 0.5 mM Cu^2+^ stress (B).

To identify the potential metabolic pathways affected by different concentrations of Cu^2+^ on strain ZSY‐33, the KEGG pathways were analysed (Figure 7). Results showed that a two‐component system, amino acid metabolism, sulfur metabolism, and pro‐energy metabolism collectively contribute to the copper‐resistant process. The most significantly enriched pathway under 0.25 mM Cu^2+^ treatment was the two‐component system. Besides, one carbon pool by folate, sulfur metabolism, and histidine metabolism pathways was significantly enriched under both 0.25 and 0.5 mM Cu^2+^ treatments. Furthermore, most of the DEGs enriched in the histidine metabolic pathway were downregulated. In the two‐component system, copper efflux was up‐regulated at both 0.25 and 0.5 mM copper stress. Phosphate assimilation, multidrug efflux, and copper efflux were up‐regulated at 0.25 mM Cu^2+^ stress. Phosphate limitation and assimilation, potassium limitation, and transport, switch motility were downregulated in the presence of 0.5 mM Cu^2+^ stress.

**FIGURE 8:**
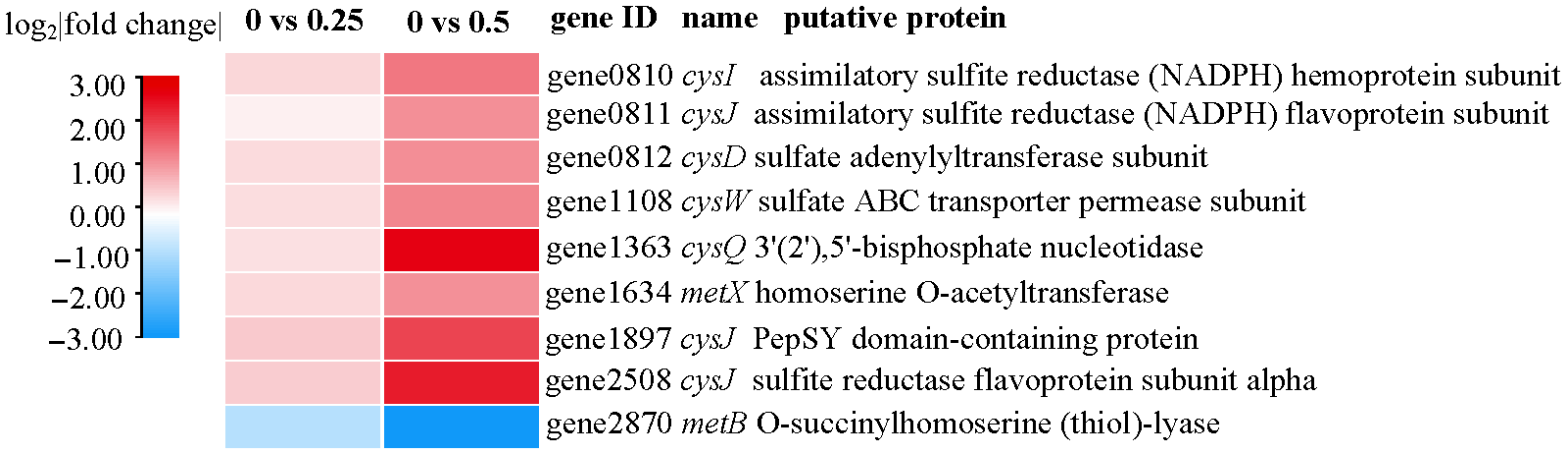
Heatmap showing the various KEGG pathways related to sulfur metabolism.

Further analysis of the genes involved in the above metabolic pathways, we found that genes exhibited different expressions in the presence of different concentrations of Cu^2+^ (Figure 8, Figure S7, and Figure S8). Genes involved in amino acid metabolism (Cysteine and methionine metabolism and Glycine, serine and threonine metabolism, etc.), sulfur metabolism, and pro‐energy metabolism (glycolysis, pyruvate metabolism, and citrate cycle) were significantly up‐ or down‐regulated in the group supplemented with 0.5 mM Cu^2+^, while the expression was not significantly changed in the group amended with 0.25 mM Cu^2+^. Genes associated with the EPS metabolic pathway including xanthan gum synthesis (such as *gumM*, *gumK*, *gumI*, *gumH*, *gumE*, *gumD*, *gumC*, and *gumK*) and synthesis of extracellular polysaccharide precursors (such as *galE*, *galM*, *gala*, and *galU*) were found to be up‐regulated in 0.25 mM Cu^2+^ treatment while down‐regulated in 0.5 mM Cu^2+^ treatment (Table S3). Genes related to sulfur metabolism, cysteine, and methionine metabolism and glutathione metabolism were significantly up‐regulated in the group supplemented with 0.5 mM Cu^2+^, including the sulfur assimilation (*cysND*, *cysC*, *cysH*, *cysJ*, and *cysI*), sulfate ABC transporter (*sbp* and *cysUWA*) and glutathione biosynthesis (*gshA* and *gshB*) (Table S4).

To validate the RNA‐seq results, five genes were selected for RT‐qPCR analysis. These included *cusA* (*gene 1982*), *copA* (*gene 2004*), *cueR* (*gene 2005*), and *copB* (*gene 2009*) which encodes copper ion efflux system, and *gshB* (*gene 1354*) relating to glutathione synthesis. The results showed that gene *cusA* and *copA* were significantly up‐regulated in the group amended with 0.5 mM Cu^2+^, and *cueR*, *copB*, and *gshB* were significantly up‐regulated in both groups amending Cu^2+^ (Figure S9). These results presented a similar pattern to the RNA sequencing data, which indicated that the expression data obtained by RNA‐seq were relatively reliable. These results demonstrated a pattern that was consistent with the RNA sequencing data, corroborating the validity of the expression data obtained from RNA sequencing.

## DISCUSSION

In the current study, a novel multiple heavy metal tolerant bacterium from *Pseudoxanthomonas* was described. The genus *Pseudoxanthomonas* was frequently found in the environment polluted by heavy metals, such as industrial wastewater (Jiang et al., 2020) and constructed wetlands (Wang et al., 2022). In this study, *P. spadix* ZSY‐33 was obtained from the Baiyangdian wetland, which is a shallow lake in North China. Due to anthropogenic activities, the mean concentrations of As, Cd, Cr, Cu, Ni, Pb, and Zn in the Baiyangdian area exceeded the environmental background values by varying degrees (Gao et al., 2013; Zhang et al., 2018). The concentration of copper in the Baiyangdian area ranged from about 0.15–0.7 mM and the background of this area was 0.34 mM (Zhang et al., 2018). Though the living environment was quite different between the natural habitat and laboratory culture, it was reasonable to hypothesize that the copper resistance of strain ZSY‐33 was consistent with the environment. Compared with *Cupriavidus gilardi*i CR3 (Huang et al., 2019), *Bacillus cereus* strain T6 (Wu et al., 2022), and *Klebsiella michiganensis* (Yan et al., 2021), the copper tolerance of strain ZSY‐33 was slightly lower. The MIC of ZSY‐33 was similar to *Pseudomonas aeruginosa* (1.6 mM) and *Providencia rettgeriand* (1.8 mM) (Hassen et al., 1998), and much higher than that of *E. coli* K12 (0.5 mM) (Hassen et al., 1998). The copper toxicity of *P. spadix* (EC_50_ = 0.5625 mM) was much lower than that determined in *Bacillus altitudinis* (EC_50_ = 5.56 mM) (Khan et al., 2022). Noteworthy, the copper resistance of strain ZSY‐33 was affected by the culture condition. In the LB medium, the MIC of copper was 1.8 mM Cu^2+^, which was much higher than that in the basic medium (Figure 2 and Figure S2). Compared with the basic medium, the LB medium was richer in organic matter and inorganic salts, especially the sulfur‐contain amino acids, which facilitated bacteria to deal with heavy metals (Yu et al., 2020).

The copper removal rate of *P. spadix* ZSY‐33 decreased with an increase in the initial Cu^2+^ concentration, which was consistence with the studies performed on *C. gilardi*i CR3 (Huang et al., 2019) and *B*. *cereus* strain T6 (Wu et al., 2022). Bioaccumulated copper ions of *P. spadix* ZSY‐33 reached saturation at a copper concentration of 0.1 mM (Figure 4B), while *P. spadix* ZSY‐33 could grow at a Cu^2+^ concentration of 0.6 mM, with delayed logarithmic growth. This indicated that *P. spadix* ZSY‐33 processed a complex system to deal with the negative impact of copper. In *P. spadix* ZSY‐33 and *C. gilardi*i CR3 (Huang et al., 2019) the detained copper was mainly concentrated inside the cells, while in *B*. *cereus* strain T6 (Wu et al., 2022), extracellular adsorption was the main channel to remove copper ions. Due to the experimental methods (EDTA‐extracted), the copper adsorbed on extracellular may be higher than the detected. Further analysis of the relationship between extracellular copper and EPS, we found that the concentration of copper affected the production of EPS (Figure 3). We proposed that high concentrations of copper promoted the production of reactive oxygen species (ROS), which causing the decrease of the cell activity and EPS (Huang et al., 2019; Li et al., 2022). Simultaneously, EPS have great effect on the bioaccumulate of extracellular copper (Figure 4). The bioaccumulate of extracellular copper decreased with the shrink of EPS. This is consistent with previous studies on *Brevundimonas diminuta* MYS6 (Rathi & Yogalakshmi, 2021), which proposed that EPS played a key role in the remove of copper. It has been shown that EPS interacted with metal ions through anionic functional groups (such as carboxylic acids, alcohols and aliphatic amine groups) and assisted the detoxification process (Li et al., 2022; Mathivanan et al., 2021a; Rathi & Yogalakshmi, 2021). Thus, in *P. spadix* ZSY‐33 we considered that copper ions were trapped by the carboxylic acids, alcohols, or aliphatic amine group in the EPS.

As for the copper accumulated in cells, we proposed that Cu^2+^ were stuck by intermediates of sulfur metabolism or amino acids metabolism. Transcriptome data showed that genes related to sulfur metabolism, cysteine, methionine metabolism, and glutathione metabolism were significantly up‐regulated in the group supplemented with 0.5 mM Cu^2+^ (Figure 7, Table S4). At the same time, the production of H_2_S was significantly inhibited by the amending of Cu^2+^ (Figure S4). It has been shown that the formation of copper sulfide (Kimber et al., 2020) or the compound with copper and glutathione (Stewart et al., 2020) could alleviate the biological toxicity of copper, while sulfhydryl groups combined with cations are important ways for Cu^2+^ detoxification (Lin et al., 2020; Yu et al., 2020; Yu & Fein, 2016; Zeng et al., 2007).

*P. spadix* ZSY‐33 possessed a complete set of cusCFBA and an incomplete set of cusCBA (absent cusF). Copper detoxification in periplasmic and cytoplasmic was facilitated by the Cus system (CusCFBA) (Bondarczuk & Piotrowska‐Seget, 2013). The Cop system of *P. spadix* ZSY‐33 including a repressor (CopY), a copper chaperone (CopZ), and two CPx‐type copper ATPases (CopA and CopB) (Solioz & Stoyanov, 2003), was responsible for cytoplasmic copper efflux, and extensively characterized as a component of copper tolerance systems (Quintana et al., 2017). Genes related to these systems were significantly up‐regulated in the treatment of both 0.25 and 0.5 mM Cu^2+^, showing a similar trend (Figure 6). This indicated that the copper pumping system was the first defence system against copper stress and was essential for the maintenance of intracellular copper homeostasis in *P. spadix* ZSY‐33 in the presence of copper. Among the copper‐resistant genes, *cueR* was the most significantly up‐regulated one in the group treated with 0.25 and 0.5 mM Cu^2+^. It was reported that copper in the cytoplasmic chaperone and plasma membrane was controlled by *cueR* (Quintana et al., 2017). Due to the high‐level expression of gene *cueR* in *P. spadix* ZSY‐33, we suspected that the *cueR* gene was an important regulator in the copper‐resistant system. In addition, active phosphate assimilation and transportation in at the present of 0.25 mM Cu^2+^ indicated that copper might be immobilized as Cu_3_(OH)_3_PO_4_ (Zhao et al., 2019) or Cu_5_(PO_4_)_2_(OH)_4_ (Do et al., 2020) crystals in the presence of soluble phosphate.

*P. spadix* ZSY‐33 activated different metabolic pathways in response to 0.25 and 0.5 mM Cu^2+^ exposure. Copper‐resistant genes were significantly up‐regulated in the group treated with 0.25 and 0.5 mM Cu^2+^, while genes related to amino acid metabolism, sulfur metabolism, and pro‐energy metabolism were significantly changed only in the treatment of 0.5 mM Cu^2+^. This indicated that different mechanisms were utilized in *P. spadix* ZSY‐33 to cope with different concentrations of copper. Under the condition of low concentration of copper, the Cop and Cus efflux systems work together to main the homeostasis of intracellular copper ions. As the concentration of copper increased, multiple metabolic pathways combined to resist copper stress. Among them, sulfur metabolism was frequently reported to be involved in the resistance of copper (Huang et al., 2019; Yan et al., 2021). Amino acids metabolic, especially the metabolic of glutathione, cysteine, and methionine, were significantly up‐regulated in the treatment of high concentrations of copper, which indicates that these metabolic pathways were responsible for *P. spadix* ZSY‐33 to deal with higher copper stress. These results were consistent with studies in yeast *Meyerozyma guilliermondii* GXDK6, which reported that the metabolism of glutathione was elevated by the copper stress (Bu et al., 2021).

**FIGURE 9:**
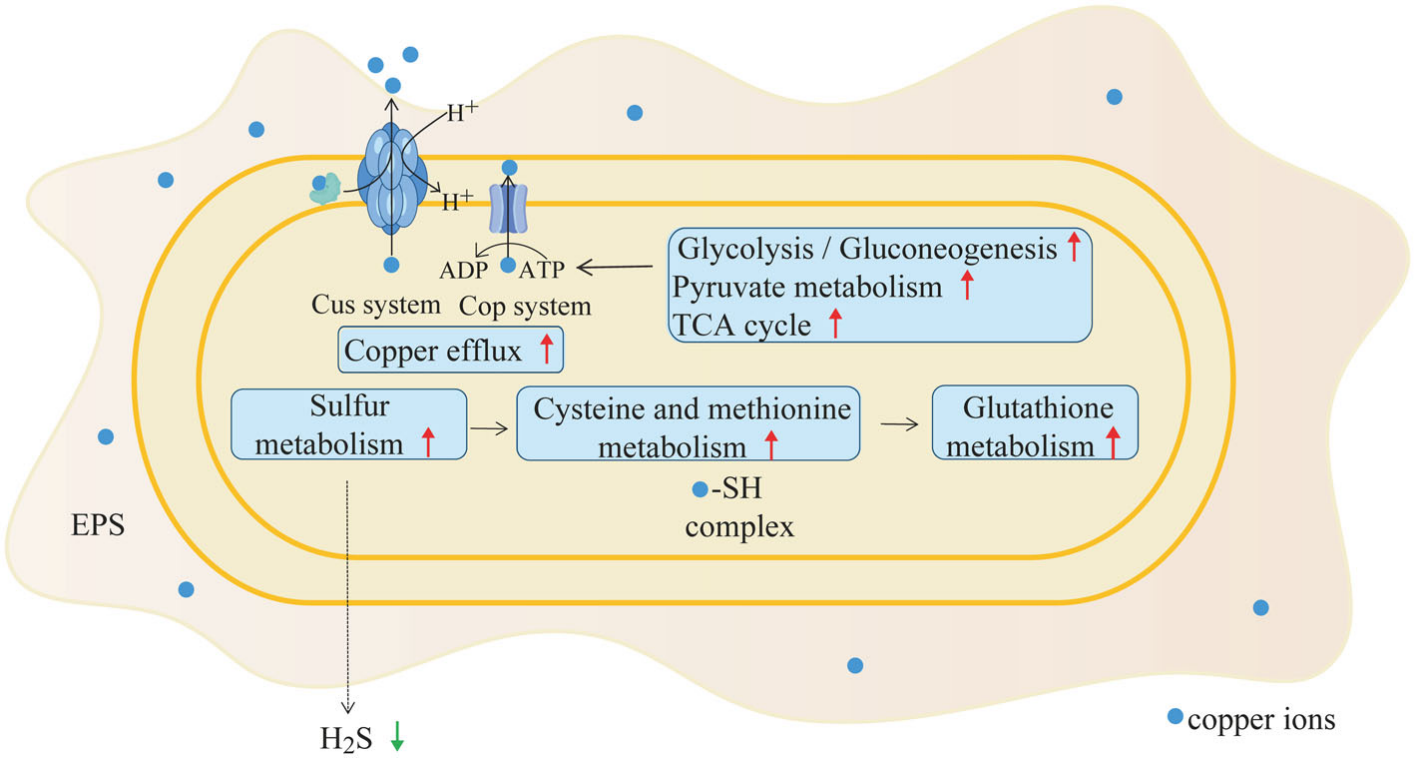
The copper resistance mechanism of *P. spadix* ZSY‐33.

Based on the analysis of the genomic and RNA‐seq data, a putative mechanism of copper stress response in strain ZSY‐33 was reconstructed (Figure 9). In sum, the copper pumping system was responsible for dealing with copper stress in the presence of copper and was an essential copper resistance strategy in strain ZSY‐33. Multiple metabolic pathways, including sulfur metabolism, amino acid metabolism (cysteine and methionine metabolism, glutathione metabolism), and pro‐energy metabolism (glycolysis/gluconeogenesis, pyruvate metabolism, and TCA cycle) were activated in response to a higher concentration of copper (0.5 mM) exposure. Sulfhydryl groups (‐SH) produced in sulfur metabolism were combined with copper to form complexes to reduce the toxicity of copper. These results showed a flexible copper resistance mechanism in strain ZSY‐33, which may be acquired from the long‐term interaction with the living environment. Despite the inconspicuous copper removal rate (Wu et al., 2022; Zhao et al., 2019), *P. spadix* ZSY‐33 was a potential candidate for heavy metal bioremediation for the tolerance of multiple heavy metals, especially in Baiyangdian wetlands.

## CONCLUSIONS

In this study, a novel copper‐resistant bacterium, *P. spadix* ZSY‐33 was isolated from a heavy metal‐contaminated wetland. The growth of strain ZSY‐33 was inhibited by a high concentration of copper, with a minimum inhibitory concentration of 0.9 mM in basic medium. The removal rate of copper decreased with the increased concentration of copper. Compared with extracellular biosorption, intracellular bioaccumulation was the main deposition mode for copper. The EPS produced by *P. spadix* ZSY‐33 was increased at a low concentration (0.25 mM) of copper and decreased at a higher concentration (0.5 mM) of copper, while the production of H_2_S was inhibited in the presence of copper. Integrative analysis of genomic and transcriptomic, the copper resistance mechanism in strain ZSY‐33 was constructed. At lower concentrations of copper, the maintenance of intracellular copper homeostasis in *P. spadix* ZSY‐33 relied on the Cus and Cop systems. As the concentration of copper increased, multiple metabolism pathways, including the metabolism of sulfur, amino acids, and pro‐energy cooperated to defend themselves against the negative impact of copper.

## AUTHOR CONTRIBUTIONS

**Hongjie Wang:** Conceptualization (lead); funding acquisition (lead); writing – original draft (equal); writing – review and editing (equal). **Siyao Zhang:** Methodology (lead); writing – original draft (equal). **Jing Zhang:** Conceptualization (supporting); funding acquisition (supporting); resources (lead); writing – original draft (equal); writing – review and editing (equal).

## CONFLICT OF INTEREST STATEMENT

The authors declare no conflicts of interest.

## Supporting information


**Data S1:** Supporting information.

## Data Availability

The NCBI GenBank accession number for the whole genome sequence (WGS) of *P. spadix* ZSY‐33 is CP095475. The accession number for the original transcriptome data is PRJNA883805.
