# Supplementary material for: The copper resistance mechanism in a newly isolated Pseudoxanthomonas spadix ZSY‐33
Source: Environ Microbiol Rep. 2023 Jun 16;15(6):484–96. doi: 10.1111/1758-2229.13163 (PMC10667631; doi:10.1111/1758-2229.13163)
Supplement: Supplementary file 1 — Data S1: Supporting information. [file EMI4-15-484-s001.docx]

**The copper resistance mechanism in a new isolated *Pseudoxanthomonas* *spadix* ZSY-33**

Hongjie Wang^1,2,3,4^, Siyao Zhang^1,3^, Jing Zhang^1,2,3,4*^

^1^ Hebei Key Laboratory of close-to-Nature restoration technology of wetlands, Hebei university, Baoding 071002

^2^ Institute of Xiong’an New Area, Hebei university, Baoding 071002

^3^ School of Eco-Environment, Hebei university, Baoding 071002

^4^ College of life science, Hebei university, Baoding 071002

^*^ Corresponding author. E-mail address: [zhjing926@126.com](mailto:zhjing926@126.com) (J. Zhang), telephone number: +86 0312 5073544


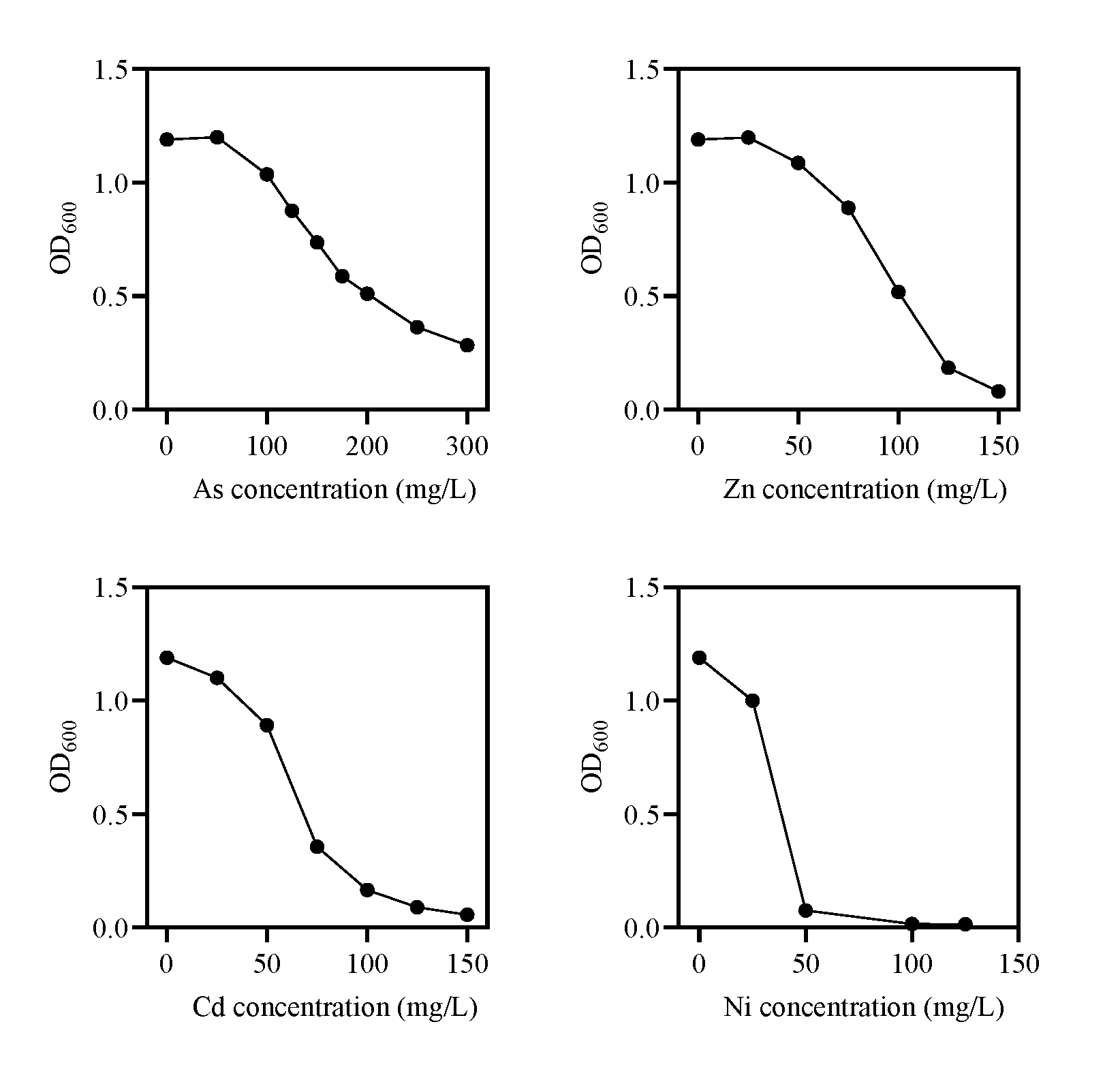


Figure S1. Multiple heavy metal resistance of ZSY-33. Minimum inhibition concentration (MIC) of arsenic, zinc, cadmium and nickel were determined in basic medium at 28 ℃ and 150 rpm for 24 h.


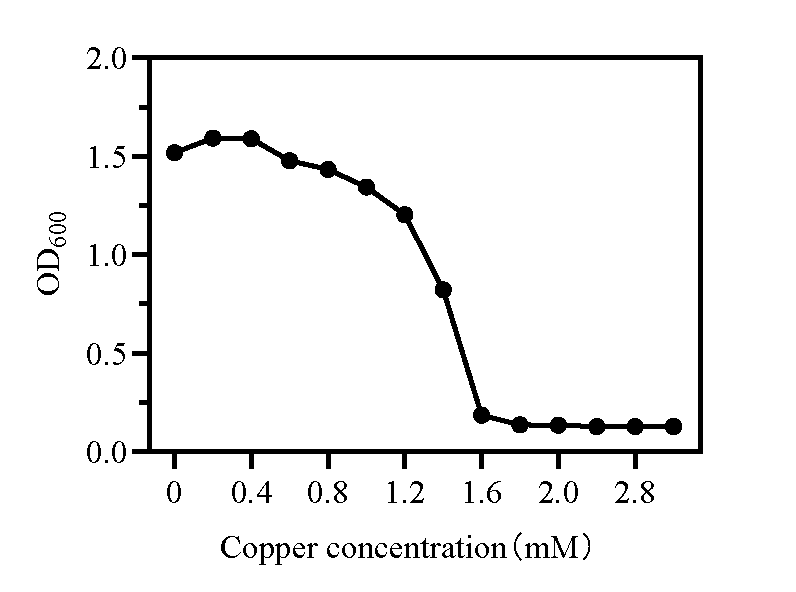


Figure S2. Minimum inhibition concentration (MIC) of copper was determined in LB at 28 ℃ and 150 rpm for 24 h.


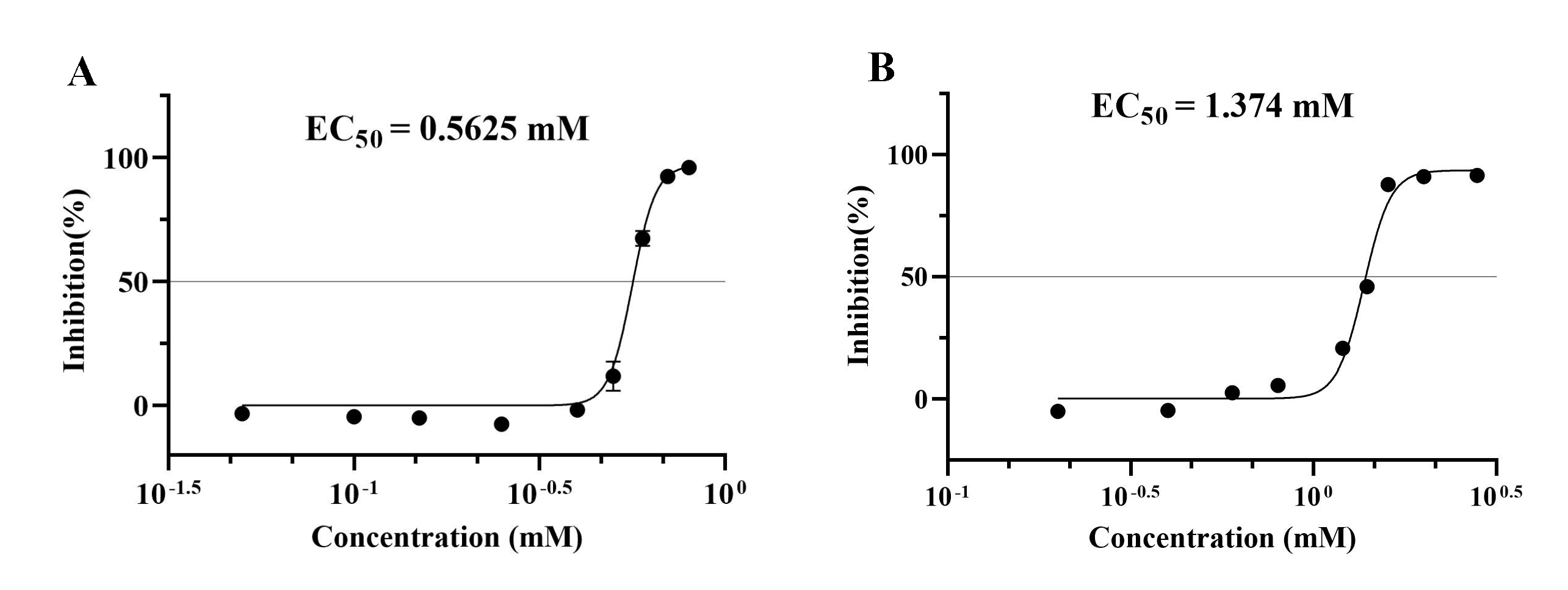


Figure S3. EC_50_ of strain ZSY-33. (A) in the Basic medium. (B) in LB medium.


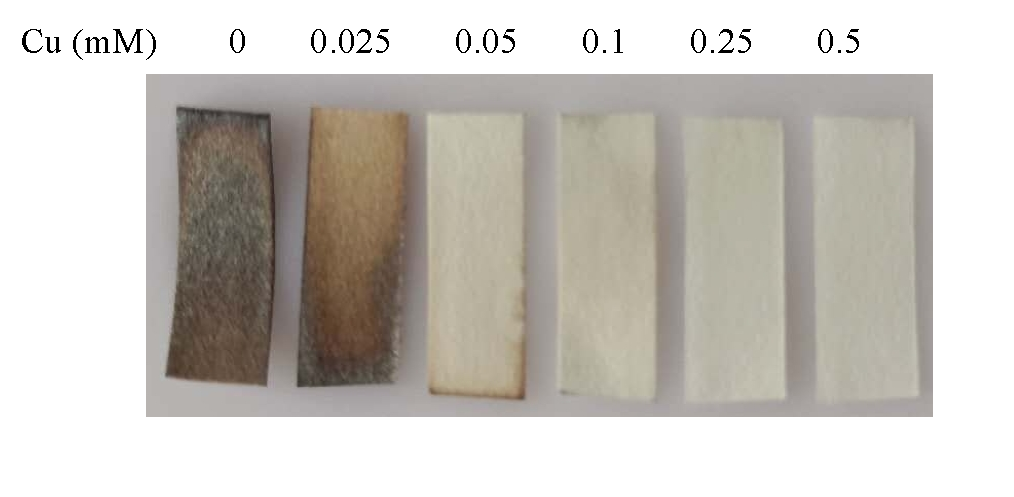


Figure S4. The production of H_2_S by strain ZSY-33 changed amending with different concentrations of copper. Cells were grown with 0, 0.025, 0.05, 0.1, 0.25 and 0.5 mM of Cu^2+^ for 24 h in basic medium.


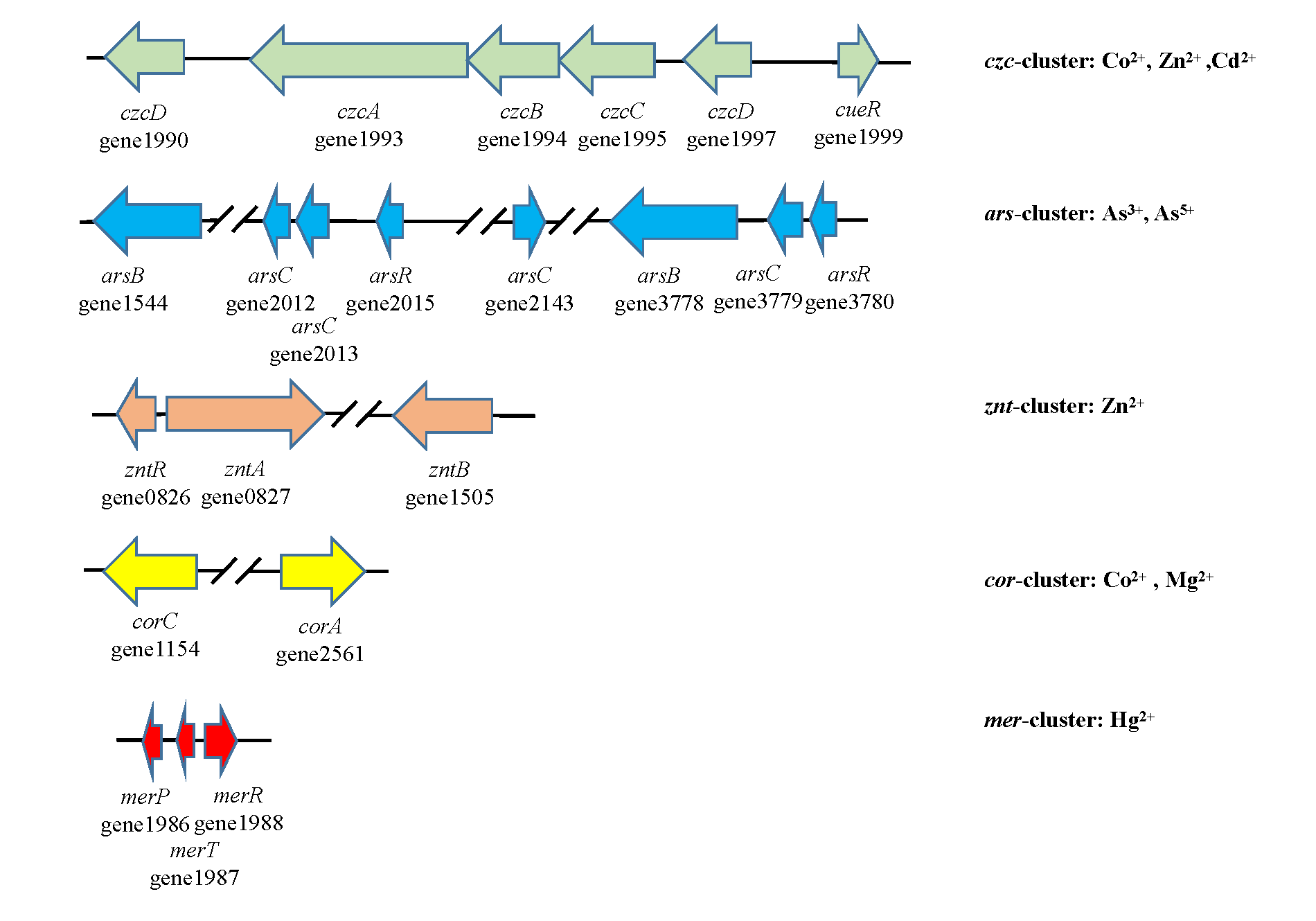


Figure S5. Orientation and product of putative gene clusters and operons involved in heavy metal resistance determinants in strain ZSY-33. Annotated genes attributed to czc is displayed in green, ars, znt, cor, mer operon were presented as blue, orange, yellow and red, respectively.


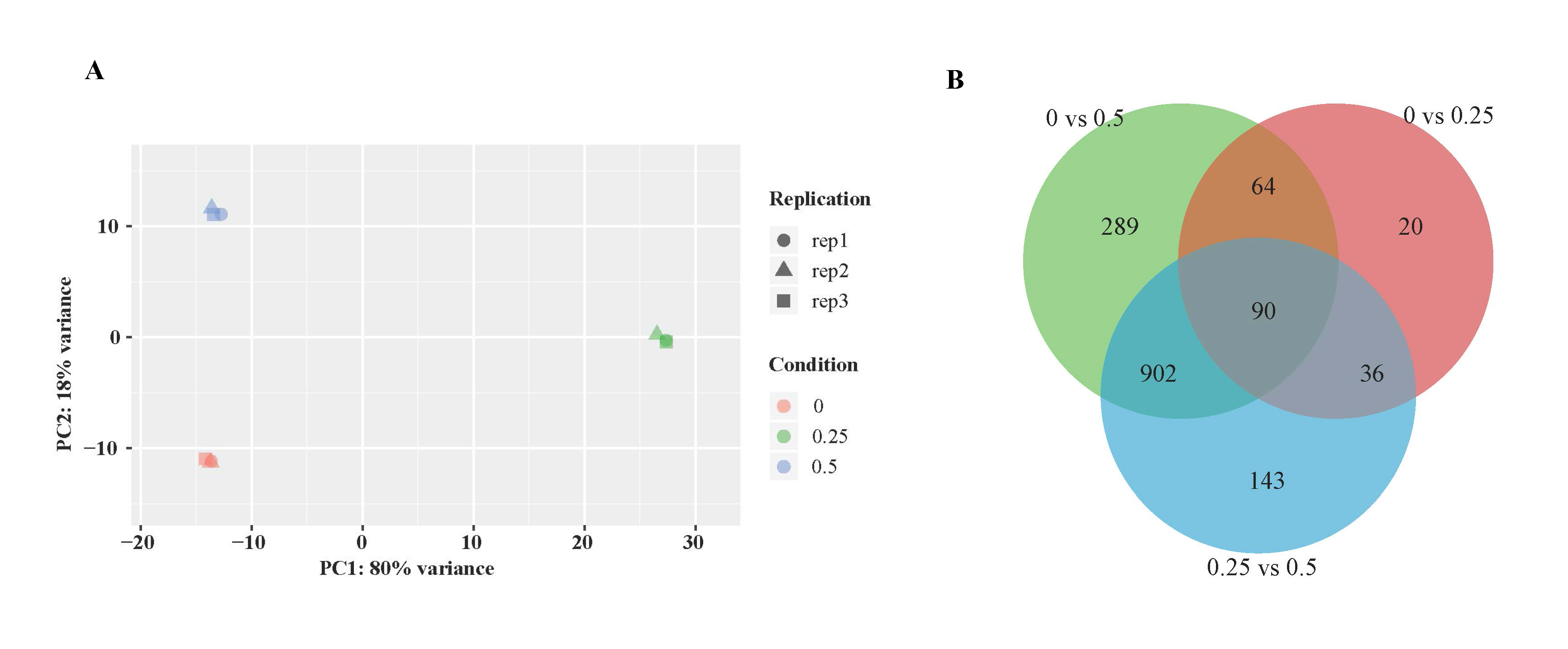


Figure S6. Transcriptome analysis of strain ZSY-33 in response to different concentrations of copper (Cu^2+^) (0, 0.25, and 0.5 mM). (A) Principal component analysis of all individual samples: 0 mM Cu^2+^, red; 0.25 mM Cu^2+^, blue; 0.5 mM Cu^2+^, green. (B) Venn diagram displaying numbers of differentially expressed transcripts in each treatment group.


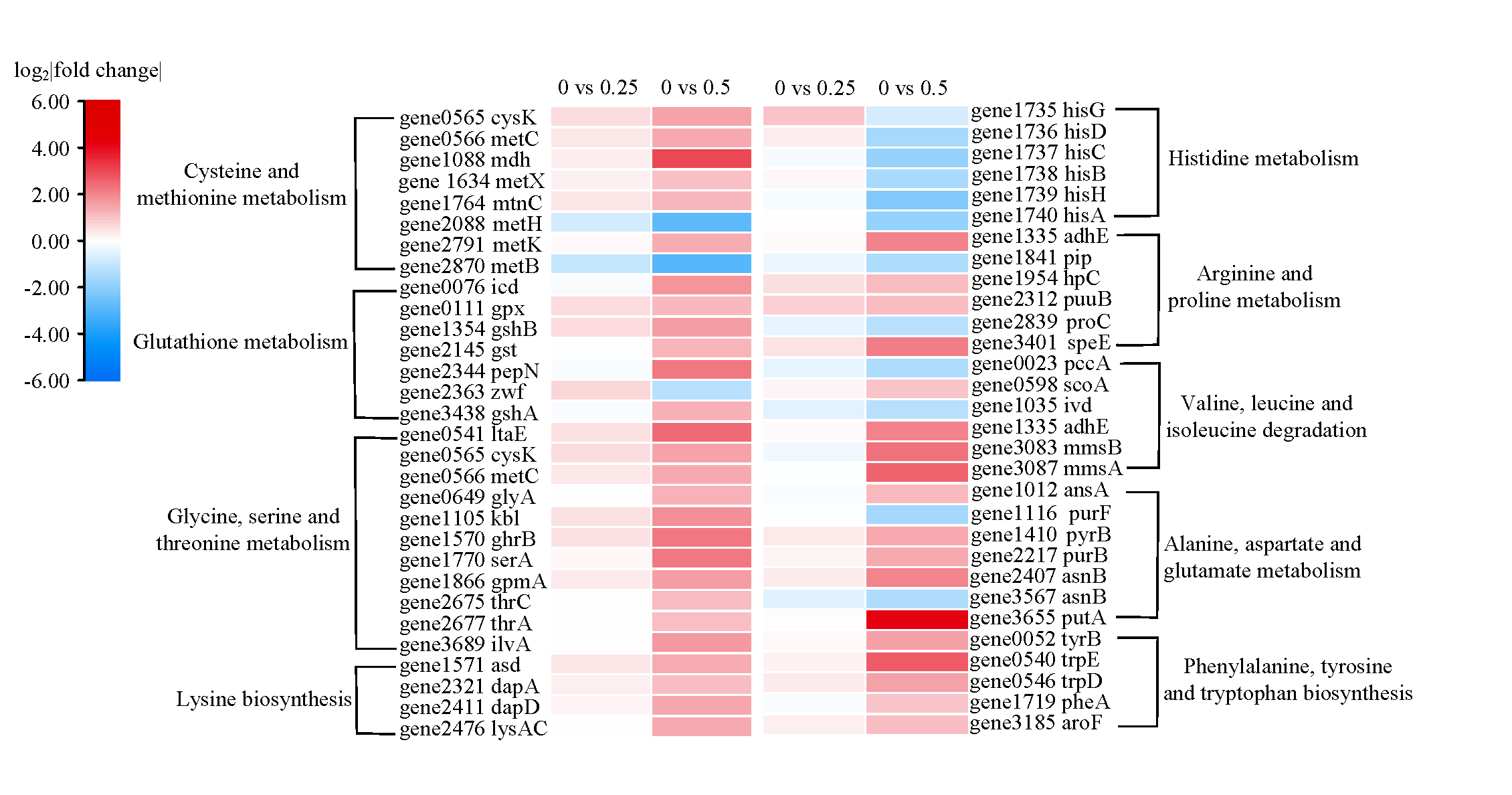


Figure S7. Heatmap showing the various KEGG pathways related to amino acid metabolism.


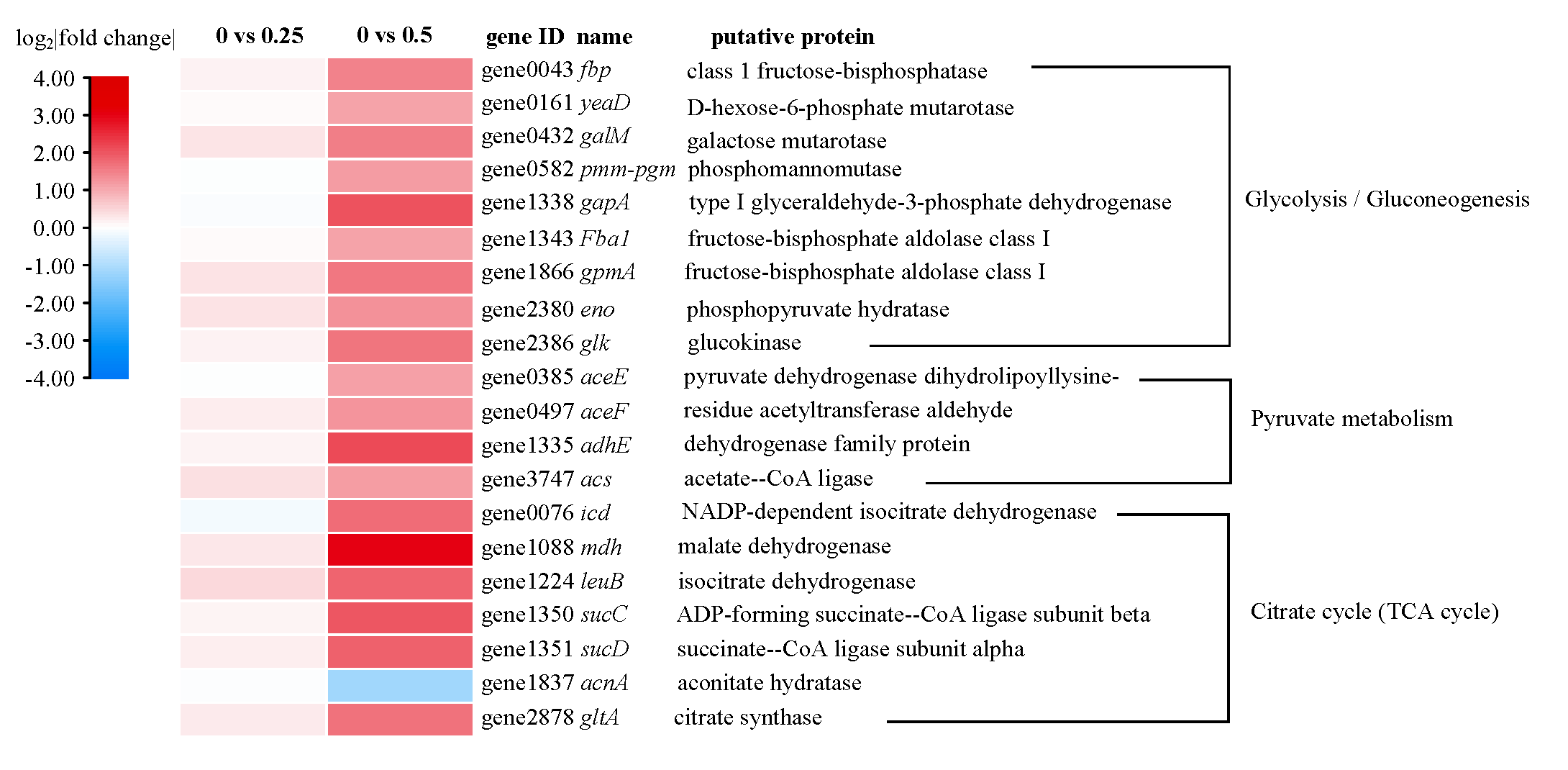


Figure S8. Heatmap showing the various KEGG pathways related to carbohydrate metabolism.


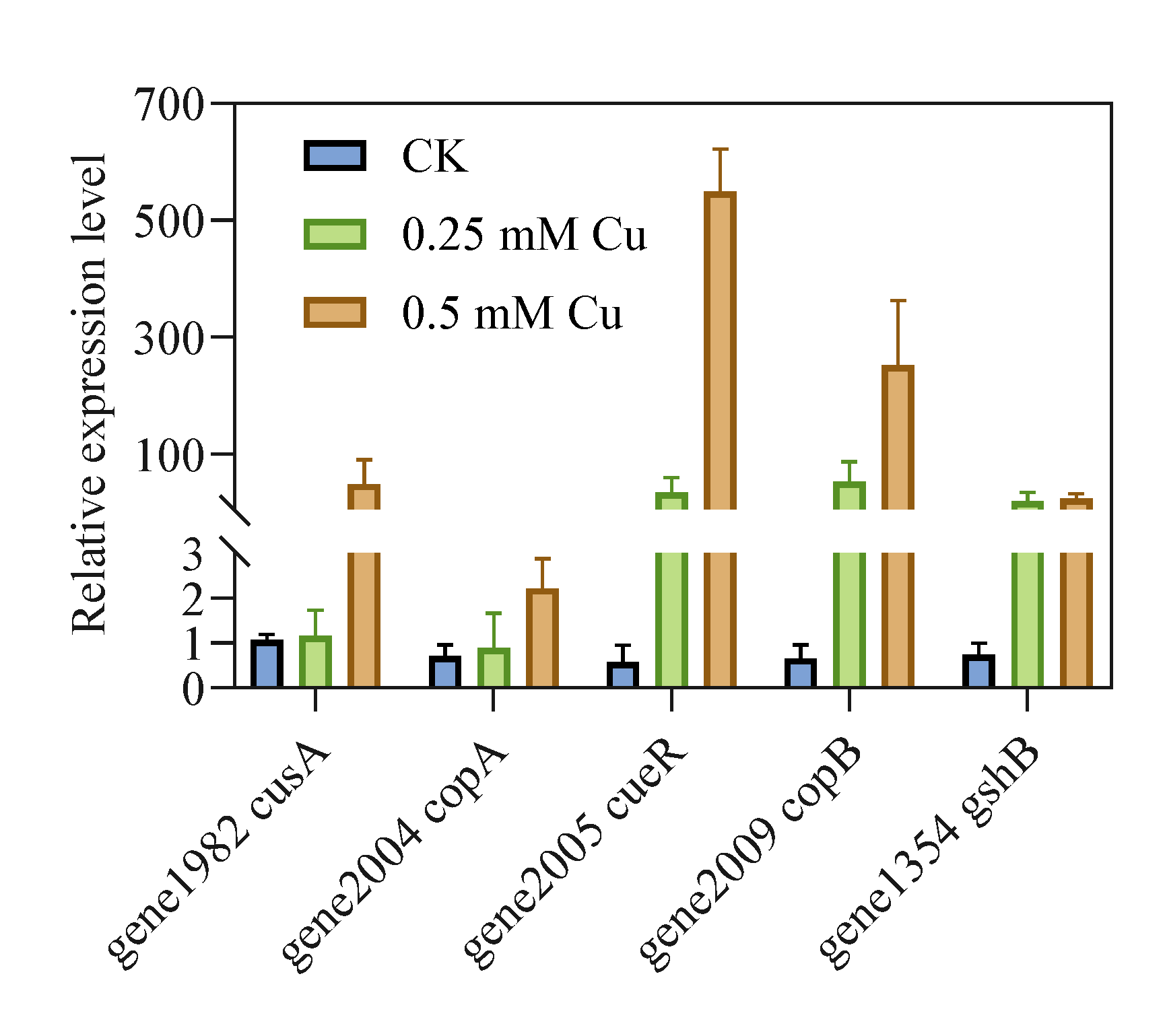


Figure S9. Quantitative determination of typical DEGs detected in the transcriptomic study.

Table S1. List of primers used for RT-qPCR in this study

| Primer | Gene Name | Description | Nuclcotide Sequence (5′-3′) |
| --- | --- | --- | --- |
| 16S-F |  |  | GGTTGCGATACTGTGAGGTGGA |
| 16S-R |  |  | GCGTTGCTGATCTGCGATTACT |
| Gene 1354-F | *gshB* | glutathione synthase | GGCCAGCTTCTCGTTGTAGT |
| Gene 1354-R |  |  | CGAGACCGAGACCGTGTTC |
| Gene 1982-F | *cusA* | efflux RND transporter permease subunit | CTCAACATACCGCCGACCAT |
| Gene 1982-R |  |  | GACAGTAGCGGTGATCCTGG |
| Gene 2004-F | *copA* | Heavy metal translocating P-type ATPase | ACAGGGTCAACGACACGATG |
| Gene 2004-R |  |  | TTCACGCTGATCGGTATCGG |
| Gene 2005-F | *cueR* | heavy metal-responsive transcriptional regulator | CTTGAGACTGCCCATGTCGT |
| Gene 2005-R |  |  | TACCAGACTGCGGATGTCCT |
| Gene 2009-F | *copB* | copper resistance protein B | GGCTTGGCTTTCGGTTTCAC |
| Gene 2009-R |  |  | TCTGCTGTTGGCCCTCAATG |

Table S2. Basic genome information of strain ZSY-33

| Genomic features | |
| --- | --- |
| Assembly size (bp) | 4,450,850 |
| Gene Average Len(bp) | 1025.69 |
| Gene Density | 0.86 |
| Chromosome number | 1 |
| Plasmid number | 0 |
| G+C content (%) | 69.22 |
| Genes (total) | 3873 |
| CDS (total) | 3813 |
| tRNA number | 54 |
| rRNA number | 6 |
| sRNA number | 104 |

| **Gene ID** | **Gene name** | **Annotated function** | **0 vs 0.25** | | | **0 vs 0.5** | | |
| --- | --- | --- | --- | --- | --- | --- | --- | --- |
|  |  |  | **log**2FC | ***p***-value | **Significant** | **log**2FC | ***p***-value | **Significant** |
| **Synthesis of extracellular polysaccharide precursors** | | | | |  |  |  |  |
| gene0262 | galE | UDP-glucose 4-epimerase GalE | 0.26 | 5.40E-01 | no | -0.78 | 8.74E-02 | no |
| gene0432 | galM | galactose mutarotase | 0.33 | 1.16E-02 | no | 1.54 | 1.73E-16 | yse |
| gene0571 | galE | NAD-dependent epimerase/dehydratase family protein | 0.13 | 3.72E-01 | no | 0.89 | 6.96E-02 | no |
| gene0939 | galA | alpha-galactosidase | 0.47 | 7.31E-03 | no | -1.00 | 1.79E-05 | yse |
| gene1814 | galU | UTP--glucose-1-phosphate uridylyltransferase GalU | 0.19 | 6.11E-02 | no | -0.36 | 1.46E-02 | no |
| **Xanthan gum synthesis** | | | | |  |  |  |  |
| gene2621 | gumM | WecB/TagA/CpsF family glycosyltransferase | 0.48 | 6.26E-03 | no | -0.31 | 1.61E-01 | no |
| gene2622 | gumK | glycosyltransferase | 0.55 | 9.02E-08 | no | -0.98 | 2.91E-10 | no |
| gene2624 | gumI | glycosyltransferase | 0.46 | 5.55E-03 | no | -1.12 | 1.57E-09 | yse |
| gene2625 | gumH | glycosyltransferase family 4 protein | 0.27 | 1.12E-01 | no | -0.45 | 2.95E-02 | no |
| gene2626 | gumE | polysaccharide biosynthesis protein GumE | 0.28 | 1.63E-01 | no | 0.32 | 1.69E-01 | no |
| gene2627 | gumD | undecaprenyl-phosphate glucose phosphotransferase | 0.49 | 1.34E-05 | no | -0.04 | 7.16E-01 | no |
| gene2628 | gumC | GumC family protein | 0.90 | 2.83E-16 | no | 0.70 | 1.19E-05 | no |
| gene2924 | gumK | UDP-glucuronate | 0.13 | 6.57E-01 | no | -0.22 | 4.54E-01 | no |

Table S3. List of genes belonging to EPS metabolism

Table S4. List of genes belonging to S metabolism

| **Gene ID** | **Gene name** | **Annotated function** | **0 vs 0.25** | | | **0 vs 0.5** | | |
| --- | --- | --- | --- | --- | --- | --- | --- | --- |
|  |  |  | **log_2_FC** | ***p*-value** | **Significant** | **log_2_FC** | ***p*-value** | **Significant** |
| **Sulfur metabolism** | | | | | | | | |
| gene0213 | tauD | TauD/TfdA family dioxygenase | -0.16 | 5.07E-01 | no | -0.29 | 4.46E-01 | no |
| gene0797 | cysK | cysteine synthase A | -0.21 | 1.08E-01 | no | 0.15 | 4.78E-01 | no |
| gene0809 | cysH | phosphoadenylyl-sulfate reductase | -0.41 | 9.69E-02 | no | 0.16 | 5.67E-01 | no |
| gene0810 | cysI | assimilatory sulfite reductase (NADPH) hemoprotein subunit | 0.26 | 3.36E-01 | no | 1.31 | 1.07E-06 | yse |
| gene0811 | cysJ | assimilatory sulfite reductase (NADPH) flavoprotein subunit | -0.01 | 9.54E-01 | no | 1.05 | 4.93E-05 | yse |
| gene0812 | cysD | sulfate adenylyltransferase subunit CysD | 0.21 | 7.78E-02 | no | 1.06 | 1.28E-08 | yse |
| gene0813 | cysNC | sulfate adenylyltransferase subunit CysN | 0.08 | 4.19E-01 | no | -0.21 | 1.78E-01 | no |
| gene1106 | sbp | sulfate ABC transporter substrate-binding protein | -0.06 | 5.06E-01 | no | -0.64 | 1.89E-05 | no |
| gene1107 | cysU | sulfate ABC transporter permease subunit CysT | 0.19 | 4.66E-01 | no | 0.12 | 6.36E-01 | no |
| gene1108 | cysW | sulfate ABC transporter permease subunit CysW | 0.19 | 3.56E-01 | no | 1.15 | 1.44E-07 | yse |
| gene1109 | cysA | sulfate/molybdate ABC transporter ATP-binding protein | 0.28 | 7.83E-02 | no | 0.06 | 7.17E-01 | no |
| gene1363 | cysQ | 3'(2'),5'-bisphosphate nucleotidase CysQ | 0.16 | 6.35E-01 | no | 2.62 | 4.65E-27 | yse |
| gene1439 | sseA | sulfurtransferase | -0.10 | 6.59E-01 | no | -0.63 | 2.90E-03 | no |
| gene1634 | metX | homoserine O-acetyltransferase | 0.24 | 7.38E-02 | no | 1.04 | 1.22E-09 | yse |
| gene1897 | cysJ | PepSY domain-containing protein | 0.41 | 1.22E-01 | no | 1.87 | 1.13E-11 | yse |
| gene2508 | cysJ | sulfite reductase flavoprotein subunit alpha | 0.38 | 4.01E-01 | no | 2.32 | 2.35E-20 | yse |
| gene2870 | metB | O-succinylhomoserine (thiol)-lyase | -1.02 | 1.46E-10 | yse | -2.96 | 3.31E-41 | yse |
| gene2871 | metX | homoserine O-succinyltransferase | -0.77 | 5.98E-05 | no | 0.53 | 1.52E-03 | no |
| gene3271 | cysK | PLP-dependent cysteine synthase family protein | 0.36 | 1.04E-01 | no | 0.55 | 5.29E-04 | no |
| **Cysteine and methionine metabolism** | | | | | | | | |
| gene0052 | tyrB | aspartate/tyrosine/aromatic aminotransferase | 0.18 | 1.17E-01 | no | 1.64 | 2.81E-25 | yse |
| gene0565 | - | pyridoxal-phosphate dependent enzyme | 0.55 | 4.48E-07 | no | 1.52 | 5.96E-22 | yse |
| gene0566 | - | cystathionine gamma-synthase | 0.38 | 1.84E-03 | no | 1.39 | 1.25E-17 | yse |
| gene1088 | mdh | malate dehydrogenase | 0.29 | 6.01E-03 | no | 3.03 | 1.31E-30 | yse |
| gene1354 | gshB | glutathione synthase | 0.58 | 3.32E-04 | no | 1.61 | 5.96E-13 | yse |
| gene1571 | asd | aspartate-semialdehyde dehydrogenase | 0.40 | 5.46E-04 | no | 1.38 | 1.03E-18 | yse |
| gene1634 | metX | homoserine O-acetyltransferase | 0.24 | 7.38E-02 | no | 1.04 | 1.22E-09 | yse |
| gene1764 | mtnC | acireductone synthase | 0.42 | 1.43E-02 | no | 1.19 | 7.35E-10 | yse |
| gene2088 | metH | homocysteine S-methyltransferase family protein | -0.81 | 2.75E-06 | no | -2.83 | 2.26E-68 | yse |
| gene2090 | metH | methionine synthase | -0.90 | 8.18E-09 | no | -2.14 | 2.31E-40 | yse |
| gene2476 | lysAC | bifunctional aspartate kinase/diaminopimelate decarboxylase | 0.01 | 8.73E-01 | no | 1.38 | 3.31E-12 | yse |
| gene2677 | thrA | bifunctional aspartate kinase/homoserine dehydrogenase I | -0.02 | 9.13E-01 | no | 1.06 | 8.19E-11 | yse |
| gene2791 | metK | methionine adenosyltransferase | 0.08 | 4.64E-01 | no | 1.35 | 1.21E-08 | yse |
| gene2870 | metB | O-succinylhomoserine (thiol)-lyase | -1.02 | 1.46E-10 | yse | -2.96 | 3.31E-41 | yse |
| gene3401 | speE | polyamine aminopropyltransferase | 0.54 | 7.64E-05 | no | 2.20 | 3.37E-13 | yse |
| gene3438 | gshA | glutamate--cysteine ligase | -0.09 | 5.11E-01 | no | 1.29 | 4.45E-14 | yse |
| **Glutathione metabolism** | | | | | | | | |
| gene0076 | icd | NADP-dependent isocitrate dehydrogenase | -0.13 | 2.06E-01 | no | 1.74 | 1.36E-29 | yse |
| gene0111 | gpx | glutathione peroxidase | 0.58 | 7.27E-04 | no | 1.14 | 1.08E-04 | yse |
| gene1354 | gshB | glutathione synthase | 0.58 | 3.32E-04 | no | 1.61 | 5.96E-13 | yse |
| gene2145 | gst | glutathione S-transferase family protein | -0.01 | 1.00E+00 | no | 1.21 | 2.92E-08 | yse |
| gene2344 | pepN | M1 family metallopeptidase | -0.09 | 4.71E-01 | no | 2.23 | 6.07E-42 | yse |
| gene2363 | zwf | glucose-6-phosphate dehydrogenase | 0.67 | 1.45E-11 | no | -1.26 | 2.52E-16 | yse |
| gene3401 | speE | polyamine aminopropyltransferase | 0.54 | 7.64E-05 | no | 2.20 | 3.37E-13 | yse |
| gene3438 | gshA | glutamate--cysteine ligase | -0.09 | 5.11E-01 | no | 1.29 | 4.45E-14 | yse |
